# Factors associated with low-density lipoprotein control in outpatients with myocardial infarction: A hospital-based study

**DOI:** 10.1371/journal.pone.0353521

**Published:** 2026-07-16

**Authors:** Anan S. Jarab, Walid Al-Qerem, Razan Mansour, Hamza Jarab, Shrouq R. Abu Heshmeh, Omar Jrab, Abdullah Elrefae, Yazid N. Al Hamarneh, Ahmad Z. Al Meslamani, Salahdein Aburuz

**Affiliations:** 1 Department of Clinical Pharmacy, Faculty of Pharmacy, Jordan University of Science and Technology, Irbid, Jordan; 2 Department of Pharmacy, Faculty of Pharmacy, Al-Zaytoonah University of Jordan, Amman, Jordan; 3 Faculty of Medicine, Jordan University of Science and Technology, Irbid, Jordan; 4 Barts and The London School of Medicine and Dentistry, Queen Mary University of London, London, Malta; 5 Trauma and Orthopedic, Northwick Park Hospital, Harrow, United Kingdom; 6 Department of Pharmacology, Faculty of Medicine and Dentistry, University of Alberta, Edmonton, Canada; 7 College of Pharmacy, Al Ain University, Abu Dhabi, United Arab Emirates; 8 Department of Pharmacology and Therapeutics, College of Medicine and Health Sciences, United Arab Emirates University, Al Ain, United Arab Emirates; University of Montenegro-Faculty of Medicine, MONTENEGRO

## Abstract

Discrepancies in factors influencing low-density lipoprotein cholesterol (LDL-C) control in patients with myocardial infarction have been documented in existing literature. Assessing LDL-C control and the associated factors is a crucial initial measure for guiding future interventions aimed at enhancing health outcomes in patients with myocardial infarction (MI). This study aimed to evaluate LDL-C control status and explore the predictors of uncontrolled LDL-C in patients with myocardial infarction. Data were collected by a research pharmacist from medical records, including sociodemographic and clinical characteristics, comorbidities, prescribed medications, and biomedical parameters such as LDL-C levels among patients attending an outpatient cardiology clinic. The validated Arabic versions of the 4-item medication adherence scale and the medication beliefs questionnaire were used to evaluate medication adherence and beliefs, respectively. Binary logistic regression was implemented to investigate the predictors of LDL-C control. The results showed most of the patients (n = 255) had uncontrolled LDL-C (76.6%). Regression results revealed that smoking (OR= 2.006; 95% CI: 1.054–3.816; P = 0.034), receiving beta blockers (OR=2.211; 95% CI: 1.024–4.775; P = 0.043), and having uncontrolled blood pressure (OR=1.940; 95% CI: 1.069–3.520; P = 0.029) were independently associated with higher odds of uncontrolled LDL-C. In conclusion, in MI patients with poor LDL-C control, lowering the risk of cardiac complications and improving health outcomes require the development of focused and efficient LDL-C level management strategies. Achieving this goal will require putting strategies like smoking cessation counselling into practice and improving BP control.

## Introduction

Myocardial Infarction (MI) is defined as “the presence of acute myocardial injury detected by abnormal cardiac biomarkers in the setting of evidence of acute myocardial ischemia” [1]. It happens when the coronary artery of the heart experiences a complete occlusion due to atherosclerosis, which results in interrupted blood flow to the heart muscle, causing damage and cell-death [1]. MI is a frequent cardiovascular disease (CVD) manifestation that entails higher risks for morbidity, mortality, and additional expenses [2–4]. A study reported that MI accounted for 208 cases per 100,000 person-years [5] and 15% of all deaths worldwide [6]. Jordan has a prevalence rate of 5.9% [7], resulting in 54.7% of all deaths each year [8]. MI puts a heavy economic burden on countries because of increased rate of hospitalizations and mortality. According to a study, MI had the highest hospitalization rate of any cardiovascular disease (CVD), at 38.8% [9].

Clinical management of MI includes reperfusion therapy, antiplatelet agents, beta-blockers, angiotensin converting enzyme inhibitors or angiotensin 2 blockers, statins, diuretics to manage symptoms, cardiac rehabilitation, and surgical intervention, if needed. However, control of risk factors, such as obesity, smoking, hypertension, hyperglycemia and dyslipidemia represents the cornerstone in MI management [10,11]

Low-density lipoprotein cholesterol (LDL-C) carries cholesterol to the arteries, and when it is present in excessive concentrations, cholesterol can build up in the blood vessel walls, exacerbating the development of plaques [12]. Furthermore, Oxidized LDL particles cause endothelial dysfunction, resulting in atherosclerosis [13]. Atherosclerosis is the main cause of death and morbidity in the world as it is the root cause of MI, stroke, and peripheral vascular disease [4]. An increased risk of CVD, including MI, has been associated with elevated LDL-C levels [14,15]. According to a meta-analysis, lowering LDL-C by 0.51 mmol/L was associated with 13% decrease in coronary death, 19% reduction in coronary revascularization, and 16% decrease in ischemic stroke [16]. Additionally, for every 1.0 mmol/L decrease in LDL-C, all-cause mortality dropped by 10%, mostly due to significant declines in deaths from coronary and other cardiovascular causes. [16]. Another study revealed that LDL-C control in MI patients lowered the risk of unstable angina by 29%, death from any cause by 28%, and death or recurrent MI by 18% [17]. Despite evidence of the beneficial impact of LDL-C control in patients with MI, uncontrolled LDL-C has been reported in several earlier studies. A previous study showed that 30.2% of MI patients had uncontrolled LDL-C levels [18]. Another study found that 52.5% of the patients had LDL-C values higher than the desired target 1–3 years post MI [19].

While previous research has been conducted, only a limited number of studies have focused specifically on LDL-C control, particularly among patients with MI, a population at high risk of further cardiovascular events if LDL-C remains uncontrolled. Additionally, discrepancies in the factors associated with LDL-C control among MI patients such as age, gender, hypertension, and diabetes have been reported in the literature [18–23]. Other studies suggested correlation between education level, smoking, statin intensity received, renin-angiotensin system inhibitors, BMI and LDL-C levels [19,23,24]. Although several studies have examined LDL-C after cardiovascular events, relatively few have focused specifically on predictors of LDL-C control among patients with MI, and evidence from Middle Eastern populations remains limited. This gap is important because patients in Jordan and similar settings may differ from those represented in Western cohorts in terms of sociodemographic characteristics, comorbidity burden, lifestyle patterns, medication beliefs, and adherence behaviors, all of which may influence LDL-C control. The present study addresses this gap by examining predictors of uncontrolled LDL-C in a Jordanian MI cohort and providing context-specific evidence to inform culturally tailored secondary prevention strategies.

## Materials and methods

### Study design, setting, and participants

At King Abdullah University Hospital (KAUH), a cross-sectional study was conducted on MI patients in the period between December 2024 and May 2025. The hospital cardiologist diagnosed the patients according to the AHA/ACC guidelines. Participants in the study had to be at least 18 years old, have a verified MI diagnosis for at least six months, be on at least one medication for MI, and sign a consent form. The study did not include patients with cognitive impairment, recent surgery, active infection or acute illness, chronic inflammatory diseases, significant hepatic or renal dysfunction, or malignancy. With the assistance of the specialized nurse, the researcher screened patients for eligibility and approached them during their hospital appointments before the cardiologist consulted them using consecutive sampling. Eligible patients were notified that participation was voluntary and would not affect usual care provided by the hospital. Additionally, they received assurances regarding their privacy and the confidentiality of the findings. A written informed consent was obtained from patients who agreed to participate in this study. Next, the researcher asked the participating patients to complete the study questionnaire, which included the medication adherence and medication beliefs questionnaires. Lastly, the researchers collected the relevant biomedical parameters from the medical records for each participant.

### Ethics approval

This work received ethical approval from Deanship of Research and KAUH’s Institutional Review Board (IRB) (Ref. 30/175/2024) on November 18, 2024. A written informed consent for participation and publication was obtained from patients who agreed to participate in this study.

### Study instruments and data collection

The researcher pharmacist gathered sociodemographic data from the participants using a specially designed questionnaire. The researcher used the medical records to collect data on comorbidities and the prescription medications. Medical records were also used to gather biomedical information such as systolic blood pressure (SBP), diastolic blood pressure (DBP), total cholesterol, triglycerides (TGs), glycosylated hemoglobin (HbA1c), LDL-C, and high-density lipoprotein cholesterol (HDL-C).Based on the ACC/AHA guidelines, the patients were labeled to have controlled LDL level if LDL-C was ≤70 mg/dL [10]. The patients were categorized to have controlled blood glucose if it HbA1c was ≤7% according to the recent ADA guidelines [11]. Patients who had SBP was ≤130 mmHg and DBP was ≤80 mmHg were considered to have controlled blood pressure according to the ACC/AHA guidelines [25]. Medication adherence was measured using the validated Arabic version of the 4-item Medication Adherence Scale [26,27], presenting four reasons for medication non-adherence including forgetfulness, carelessness, discontinuing medication when improving, and stop taking medication when feeling worse. Those who had three or more “yes” answers were considered to have low adherence, those who gave one or two “yes” answers as moderate, and those who gave four “no” answers as high. This scale assessed general medication adherence and was not specific to statin therapy. Medication-related attitudes were evaluated in terms of medication necessity (5 items) and medication concerns (5 items) using the validated Arabic version of the particular attitudes about Medicines Questionnaire (BMQ) [28,29].

### Sample size calculation

The minimal sample size needed to do binary logistic regression was calculated using the formula 50 + 8P, where p is the number of predictors. The study’s initial goal was to assess how the 32 variables related to LDL-C control. Consequently, 306 was the bare minimum sample size needed. The final model included 16 predictor parameters, corresponding to approximately 20.8 observations per parameter.

### Statistical analysis

Data analyses were performed using the Statistical Package for the Social Sciences (SPSS, version 28, Illinois, New York, USA). Frequencies and percentages were used to report categorical variables. Based on Q-Q plot test of normality, continuous variables were not normally distributed and displayed as medians with interquartile ranges (IQRs). Pearson’s correlation and Chi-square tests to ascertain the correlation between various factors and LDL-C status. The variables included in the bivariate analysis were all demographic variables, medications with prevalence above 5%, necessity and concerns scores, adherence level, HbA1c and blood control status. A binary logistic regression analysis was then performed to identify factors independently associated with uncontrolled LDL-C. Variables with p-values below 0.25 in the bivariate analysis were considered eligible for inclusion in the multivariable model. Before model fitting, multicollinearity was assessed by calculating variance inflation factors (VIFs), and all VIF values were below 3, indicating no evidence of multicollinearity. Model performance was further evaluated by examining overall fit and calibration using the Nagelkerke R², and the Hosmer-Lemeshow goodness-of-fit test, while discriminatory ability was assessed using the area under the receiver operating characteristic curve.

## Results

The study recruited 333 out of 420 invited patients with MI, resulting in a response rate of 79.3%. The median age was 58 years (IQR: 53–65). The participants were mostly male (75.4%), married (95.8%), physically inactive (82.2%), had poor income (68.2%), low education (64.7%), were obese (45.6%), and had a family history of cardiovascular disease (69.0%). Table 1 displays the sociodemographic details and their distribution according to LDL-C control among the study participants. The most common comorbid diseases included hypertension (92.2%), diabetes (54.1%), and dyslipidemia (47.7%).

**Table 1:** Sociodemographic characteristics of the study patients overall and stratified by LDL-C control status (n = 333).

Characteristics		All participants n = 333	LDL status	
			Controlled n=78 (23.4%)	Uncontrolled n=255 (76.6%)
		Median (IQR) or frequency (%)		
Age		58 (53-65)	58 (54-66)	58 (52-64)
Gender	Male	251 (75.4%)	60 (76.9%)	191 (74.9%)
	Female	82 (24.6%)	18 (23.1%)	64 (25.1%)
Marital status	Other*	14 (4.2%)	2 (2.6%)	12 (4.7%)
	Married	318 (95.8%)	75 (97.4%)	243 (95.3%)
Educational level	Low (less than college/university)	214 (64.7%)	56 (71.8%)	158 (62.5%)
	High (college/university)	117 (35.3%)	22 (28.2%)	95 (37.5%)
Currently employed	No	212 (63.9%)	58 (74.4%)	154 (60.6%)
	Yes	120 (36.1%)	20 (25.6%)	100 (39.4%)
Monthly income	Less than 500 JDs	223 (67.0%)	58 (74.4%)	165 (64.7%)
	More than 500 JDs	110 (33.0%)	20 (25.6%)	90 (35.3%)
Residency	Countryside	141 (42.5%)	38 (49.4%)	103 (40.4%)
	City	191 (57.5%)	39 (50.6%)	152 (59.6%)
Performing regular physical activity	No	272 (82.2%)	70 (89.7%)	202 (79.8%)
	Yes	59 (17.8%)	8 (10.3%)	51 (20.2%)
Smoking	No	192 (57.7%)	57 (73.1%)	135 (52.9%)
	Yes	141 (42.3%)	21 (26.9%)	120 (47.1%)
BMI*(kg/m^2^)	Less than 25	51 (15.3%)	13 (16.7%)	38 (14.9%)
	25-29.9	130 (39%)	25 (32.1%)	105 (41.2%)
	30 or more	152 (45.6%)	40 (51.3%)	112 (43.9%)
Family history of CVD*	No	103 (31.0%)	30 (38.5%)	73 (28.7%)
	Yes	229 (69.0%)	48 (61.5%)	181 (71.3%)
Beliefs about medication	Concern	10 (8-13)	9 (8-12)	10 (8-13)
	Necessity	18 (13-21)	18 (12-21)	18 (13-22)
Adherence status	High	151 (45.3%)	34 (43.6%)	117 (45.90%)
	Low			

*BMI: Body Mass Index, CVD: Cardiovascular disease

As shown in Table 2, the median (IQR) LDL-C was 2.38 mmol/L (1.80–3.08) and most of the patients had uncontrolled LDL-C (76.6%). Furthermore, 47.7% and 42.0% of the patients had uncontrolled BP and HbA1c, respectively, with a higher percentage of uncontrolled BP and HbA1c among the uncontrolled LDL-C group (50.6% vs. 38.5% and 42.4% vs. 41.0%, respectively).

**Table 2:** Biomedical profiles of the study patients overall and stratified by LDL-C control status (n = 333).

Variables		All participants n = 333	LDL status	
			Controlled n=78 (23.4%)	Uncontrolled n=255 (76.6%)
		Median (IQR) or frequency (%)		
LDL-C (mmol/L) *		2.38 (1.80-3.08)	1.46 (1.19-1.65)	2.64 (2.19-3.39)
SBP*		125 (117-138)	122 (115-137)	127 (118-139)
DBP*		80 (72-85)	79 (70-80)	80 (74-85)
Blood pressure status	Controlled	174 (52.3%)	48 (61.5%)	126 (49.4%)
	Uncontrolled	159 (47.7%)	30 (38.5%)	126 (50.6%)
HbA1c		6.22 (5.70-7.70)	6.60 (5.90-7.85)	6.10 (5.60-7.67)
HbA1c status	Controlled	193 (58.0%)	46 (59.0%)	147 (57.6%)
	Uncontrolled	140 (42.0%)	32 (41.0%)	108 (42.4%)

* LDL-C: Low Density Lipoprotein, SBP: Systolic Blood Pressure, DBP: Diastolic Blood Pressure.

For LDL-C, 1 mmol/L = 38.67 mg/dL; therefore, 70 mg/dL is approximately 1.8 mmol/L.

Table 3 shows that the median number of medications prescribed for MI and the total prescribed medications were 5 (IQR: 4–6) and 7 (IQR: 6–9), respectively. Of the 318 individuals treated with statins, 72.7% were treated with high-intensity statins and 22.8% with moderate-intensity statins. 94.0% of the patients received antiplatelets, particularly 91.3% received aspirin, 48.9% received clopidogrel., and 46.2% received both. Beta blockers were the most often given antihypertensive drugs (82.9%), whereas metformin was the most often recommended antidiabetic drug (36.3%). Most of the patients reported high (45.3%) and moderate adherence (45.3%). While only (11.1%) reported low adherence. According to the BMQ, the most reported medication necessities were “My medicines protect me from becoming worse” (84.3%) and “Without my medicines, I would be very sick” (71.1%). In contrast, the least necessity was “My health in the future will depend on my medicines” (36%). The impact of the prescribed medication on daily activities (14.1%) was the least common concern, while the long-term effects of my medicines” (34.2%) was the most prevalent. The median scores for medication necessity and concerns were 18 (IQR: 13–21) and 10 (IQR: 8–13), respectively.

**Table 3:** Medication history, adherence, and beliefs about medicines among enrolled patients overall and stratified by LDL-C control status (n = 333).

Variables		All participants n = 333	LDL status	
			Controlled n=78 (23.4%)	Uncontrolled n=255 (76.6%)
		**Median (IQR) or frequency (%)**		
Number of MI medications		5 (4-6)	5 (4-7)	5 (4-6)
Total number of medications		7 (6-9)	8 (6-10)	7 (6-9)
	Once	52 (15.7%)	8 (10.4%)	44 (17.3%)
	Twice	191 (57.5%)	43 (55.8%)	148 (58%)
	Thrice or more	89 (26.8%)	26 (33.8%)	63 (24.7%)
Medications for Lipid control				
Statins	No	15 (4.5%)	2 (2.6%)	13 (5.1%)
	Moderate intensity	76 (22.8%)	19 (24.4%)	57 (22.4%)
	High intensity	242 (72.7%)	57 (73.1%)	185 (72.5%)
Fibrates	No	314 (94.3%)	76 (97.4%)	238 (93.3%)
	Yes	19 (5.7%)	2 (2.6%)	17 (6.7%)
Medications for Blood Pressure control				
Beta blockers	No	57 (17.1%)	17 (21.8%)	40 (15.7%)
	Yes	276 (82.9%)	61 (78.2%)	215 (84.3%)
ACEIs*	No	220 (66.1%)	52 (66.7%)	168 (65.9%)
	Yes	113 (33.9%)	26 (33.3%)	87 (34.1%)
ARBs*	No	218 (65.5%)	48 (61.5%)	170 (66.7%)
	Yes	115 (34.5%)	30 (38.5%)	85 (33.3%)
CCBs*	No	241 (72.4%)	52 (66.7%)	189 (74.1%)
	Yes	92 (27.6%)	26 (33.3%)	66 (25.9%)
Diuretics	No	198 (59.5%)	36 (46.2%)	162 (63.5%)
	Yes	135 (40.5%)	42 (53.8%)	93 (36.5%)
Vasodilators	No	261 (78.4%)	59 (75.6%)	202 (79.2%)
	Yes	72 (21.6%)	19 (24.4%)	53 (20.8%)
Medications for Blood Glucose				
Metformin	No	212 (63.7%)	48 (61.5%)	164 (64.3%)
	Yes	121 (36.3%)	30 (38.5%)	91 (35.7%)
Sulfonylurea	No	286 (85.9%)	69 (88.5%)	217 (85.1%)
	Yes	47 (14.1%)	9 (11.5%)	38 (14.9%)
DPP-4 inhibitors*	No	306 (91.9%)	75 (96.2%)	231 (90.6%)
	Yes	27 (8.1%)	3 (3.8%)	24 (9.4%)
Insulin	No	273 (82%)	57 (73.1%)	216 (84.7%)
	Yes	60 (18%)	21 (26.9%)	39 (15.3%)
Antiplatelets				
Antiplatelet	No	20 (6.0%)	6 (7.7%)	14 (5.5%)
	Yes	313 (94.0%)	72 (92.3%)	241 (94.5%)
Aspirin	No	29 (8.7%)	7 (9.0%)	22 (8.6%)
	Yes	304 (91.3%)	71 (91.0%)	233 (91.4%)
Clopidogrel	No	170 (51.1%)	39 (50%)	131 (51.4%)
	Yes	163 (48.9%)	39 (50.0%)	124 (48.6%)
Dual antiplatelet therapy	No	179 (53.8%)	40 (51.3%)	139 (54.5%)
	Yes	154 (46.2%)	38 (48.7%)	116 (45.5%)
Anticoagulants				
Warfarin	No	322 (96.7%)	74 (94.9%)	248 (97.3%)
	Yes	11 (3.3%)	4 (5.1%)	7 (2.7%)
Anti-arrhythmic drugs				
Amiodarone	No	323 (97%)	74 (94.9%)	249 (97.6%)
	Yes	10 (3%)	4 (5.1%)	6 (2.4%)
Digoxin	No	322 (96.7%)	73 (93.6%)	249 (97.6%)
	Yes	11 (3.3%)	5 (6.4%)	6 (2.4%)
Other medications				
PPIs*	No	69 (20.7%)	12 (15.4%)	57 (22.4%)
	Yes	264 (79.3%)	66 (84.6%)	198 (77.6%)

*MI: Myocardial Infarction, ACEIs: Angiotensin Converting Enzyme Inhibitors, ARBs: Angiotensin Receptor Blockers, CCBs: Calcium Channel Blockers, DPP-4 inhibitors: Dipeptidyl Peptidase-4 inhibitors, PPIs: Proton Pump inhibitors

The bivariate analysis included all demographic variables listed in Table 1, all the medications with frequency of usage above 5% listed in Table 3 and HbA1c and blood pressure control status. According to bivariate analysis, LDL status was linked to smoking (P = 0.002), engaging in regular physical activity (P = 0.046), having a job (P = 0.027), taking diuretics (P = 0.005), taking insulin (P = 0.017), and taking all the prescribed drugs (P = 0.002). All variables with bivariate p-values below 0.20 that are listed in Table 4 were included in the logistic regression. The logistic regression revealed that smoking (OR= 2.006; 95% CI: 1.054–3.816; P = 0.034), receiving beta blockers (OR=2.211; 95% CI: 1.024–4.775; P = 0.043), and uncontrolled blood pressure (OR=1.940; 95% CI: 1.069–3.520; P = 0.029) were associated with higher odds of uncontrolled LDL-C. The logistic regression model showed moderate discrimination, with an area under the curve of 0.740 (SE = 0.032, 95% CI: 0.677–0.804; p < 0.001). Model fit was acceptable, with a Nagelkerke R² of 0.192. The Hosmer-Lemeshow test was not significant (χ² = 8.351, df = 8, p = 0.400), indicating adequate calibration

**Table 4:** Factors associated with uncontrolled LDL-C in binary logistic regression.

Variable	Comparison	Crude OR (95% CI)	p-value	Adjusted OR (95% CI)	p-value
Educational level	College/university vs less than college/university	1.530 (0.879-2.666)	0.133	1.418 (0.730-2.755)	0.303
Currently employed	Yes vs No	1.883 (1.068-3.320)	0.029	1.193 (0.601-2.368)	0.615
Residency	City vs countryside	1.438 (0.862-2.399)	0.164	1.221 (0.683-2.183)	0.501
Performing regular physical activity	Yes vs No	2.209 (0.999-4.884)	0.050	1.460 (0.586-3.640)	0.416
Smoking	Yes vs No	2.413 (1.382-4.213)	0.002	2.006 (1.054-3.816)	0.034
Family history of CVD	Yes vs No	1.550 (0.911-2.635)	0.106	1.427 (0.770-2.643)	0.258
Arrhythmia	Yes vs No	0.518 (0.237-1.136)	0.101	0.474 (0.192-1.171)	0.106
Total number of medications	Per additional medication	0.848 (0.764-0.942)	0.002	0.921 (0.777-1.091)	0.341
Beta blockers	Yes vs No	1.498 (0.794-2.826)	0.212	2.211 (1.024-4.775)	0.043
CCBs	Yes vs No	0.698 (0.404-1.208)	0.199	1.210 (0.597-2.450)	0.597
Diuretics	Yes vs No	0.492 (0.295-0.822)	0.007	0.642 (0.327-1.257)	0.196
Insulin	Yes vs No	0.490 (0.267-0.898)	0.021	0.699 (0.297-1.645)	0.412
PPIs	Yes vs No	0.632 (0.319-1.249)	0.187	0.670 (0.281-1.600)	0.367
Blood pressure status	Uncontrolled vs controlled	1.638 (0.976-2.750)	0.062	1.940 (1.069-3.520)	0.029
HbA1c	Per 1% increase	0.876 (0.760-1.010)	0.069	0.925 (0.766-1.116)	0.414
Medication-related concerns	Per 1-point increase	1.054 (0.985-1.126)	0.126	1.055 (0.976-1.140)	0.179

*CVD: Cardiovascular disease, CCBs: Calcium Channel Blockers, PPIs: Proton Pump Inhibitors. ORs above 1 indicate higher odds of uncontrolled LDL-C for the first category listed in the comparison/reference column.

## Discussion

Previous research has demonstrated the correlation between uncontrolled LDL-C levels and the incidence of MI in individuals without a history of heart attack [30,31]. Furthermore, it has been found that failure to maintain LDL-C levels within the target range after an MI can increase risk of recurrent MIs and other cardiac complications [32]. High LDL-C levels also contribute to inflammation and oxidative stress, exacerbating damage to the heart tissue following an MI [33].

In the present study, lipid control status was far below expectations, with approximately 76.6% of participants showing uncontrolled LDL-C levels. Similarly, a study conducted in South Africa revealed that 87.3% of patients presenting with acute MI (AMI) had hyperlipidemia [34]. In Portugal, a study reported that 85% of patients who had significant coronary artery disease (CAD) had dyslipidemia. However, only 20.3% had high LDL-C levels [35]. Conversely, a study conducted in Libya found a relatively lower prevalence of hypercholesterolemia and hypertriglyceridemia, with approximately one-third of acute MI patients being affected [36]. Research conducted in the United States revealed that one-third of participants failed to achieve the target LDL-C levels (<100 mg/dl) six months after experiencing MI [37]. In China, before undergoing percutaneous coronary intervention, only a small percentage of patients with acute coronary syndrome (ACS) had high LDL-C levels (10.9%). Surprisingly, after one year of intervention, the prevalence decreased even further, reaching 3.25% [38]. These studies collectively highlight the concerning trends in LDL-C control status among patients with cardiovascular conditions in various countries, indicating a need for improved management and preventive measures. Compared with studies in other post-MI and cardiovascular outpatient settings, the prevalence of uncontrolled LDL-C in the present Jordanian cohort appears high. In the Italian EYESHOT Post-MI registry, 52.5% of patients evaluated 1–3 years after MI had LDL-C ≥ 70 mg/dL [19], whereas 76.6% of patients in our study were above the LDL-C target. Similarly, low target attainment has been reported in Spain, where only 34.0% of outpatients with previous MI achieved LDL-C < 70 mg/dL in 2018 [21]. In contrast, a United States AMI registry reported that approximately one-third of patients with initially elevated LDL-C failed to reach a less stringent LDL-C goal of <100 mg/dL at 6 months [37]. These differences may be explained by variation in LDL-C thresholds, timing of lipid assessment after MI, patient adherence, treatment intensification, access to lipid-lowering therapy, and follow-up systems across healthcare settings. Beyond differences in the prevalence of uncontrolled LDL-C, variation across studies may also reflect differences in the factors associated with LDL-C control. In the present study, smoking, beta-blocker use, and uncontrolled blood pressure were associated with uncontrolled LDL-C, although the association with beta-blocker use should be interpreted cautiously because of the possibility of confounding by indication. The associations with smoking and uncontrolled blood pressure support the concept that poor lipid control may cluster with other modifiable cardiovascular risk factors. Previous post-MI and cardiovascular outpatient studies have similarly emphasized that LDL-C target attainment is influenced not only by baseline risk, but also by treatment intensity, adherence or persistence with lipid-lowering therapy, follow-up, and timely treatment intensification [19,21,37,39]. Therefore, the high rate of uncontrolled LDL-C observed in this cohort should be interpreted as part of a broader secondary-prevention gap rather than as an isolated lipid-management issue. These findings support the need for structured post-MI follow-up programs that integrate LDL-C monitoring, statin-specific adherence assessment, smoking cessation, blood pressure optimization, and treatment intensification when LDL-C targets are not achieved.

Smoking was correlated with uncontrolled LDL-C levels in this research study. This finding aligns with the evidence reported in previous literature, which consistently indicated a strong association between cigarette smoking and dyslipidemia [40,41]. The mechanism by which cigarette smoking influences lipid levels has been a subject of investigation in earlier research. One prevailing hypothesis points to nicotine absorption as the instigator of a cascade of hormonal responses. Specifically, the secretion of catecholamines, cortisol, and growth hormones is triggered, setting in motion the activation of adenyl cyclase in adipose tissue. This biochemical chain reaction results in the breakdown of stored TG and the subsequent release of free fatty acids, leading to hyperlipidemia [42]. Considering the implications of dyslipidemia for cardiovascular health, healthcare providers must prioritize the implementation of smoking cessation counseling and emphasize the critical significance of quitting smoking. Additionally, it has been found that the use of mobile health tools, such as smartphones and patient monitoring devices, can help individuals quit smoking, which healthcare providers could consider supporting patients in quitting [43].

Beta-blocker use was associated with higher odds of uncontrolled LDL-C in this study. However, this association should be interpreted cautiously and should not be considered evidence that beta-blockers caused poor LDL-C control. Although some beta-blockers, particularly older non-selective agents, may adversely affect lipid parameters by increasing triglycerides and lowering HDL-C, their effects on LDL-C are inconsistent and vary by agent [44–46]. In addition, patients receiving beta-blockers after MI may differ clinically from non-users, particularly with respect to MI severity, residual ischemia or angina, heart failure, arrhythmias, left ventricular function, and overall cardiometabolic risk. Therefore, the observed association may reflect confounding by indication rather than a direct pharmacologic effect. Because this study was cross-sectional and did not capture the timing of beta-blocker initiation relative to LDL-C measurement, beta-blocker type, dose, duration, MI severity, left ventricular function, or residual ischemia, causality cannot be inferred. Future longitudinal studies with detailed treatment and clinical-severity data are needed to clarify this relationship [46].

Consistent with findings from Nigeria [47], Iraq [48], and Spain [49], poor BP control was correlated with poor LDL-C control. It is well-established that hypertension and dyslipidemia frequently coexist, with thiazide diuretics used for hypertension treatment, potentially exacerbating hyperlipidemia by inducing potassium or sodium depletion [50]. Additionally, another research linked the extreme prevalence of dyslipidemia in patients with high blood pressure to adiposity [51]. Considering these findings, it is imperative to direct special attention towards managing dyslipidemia in hypertensive patients, especially those with a history of MI. To enhance lipid control status and mitigate the detrimental impact of poor lipid management on patients’ health outcomes, healthcare professionals should prioritize achieving the target BP levels and ensure better overall health for patients in this population.

Compared to previous research, the current study specifically targeted patients with MI, a high-risk group for cardiovascular events due to poor LDL-C control. Additionally, this study examined a broader range of factors influencing LDL-C control, including medication adherence, medication beliefs, and various demographic, disease, medication, and biomedical variables. Furthermore, to the best of our knowledge, this study is the first to provide insights into the factors influencing LDL-C control among MI patients in the Middle East context. Lastly, the findings of this study could help narrow down and refine the factors associated with LDL-C control in this patient group. Collectively, these factors contribute to the unique value of this study and its contribution to existing literature. Although 72.7% of patients receiving statins were prescribed high-intensity statin therapy, prescription of high-intensity statins does not necessarily confirm adequate statin exposure or consistent use. The adherence scale used in this study assessed general medication adherence rather than statin-specific adherence. Therefore, statin non-adherence, discontinuation, or inconsistent use may have contributed to the high prevalence of uncontrolled LDL-C observed in this cohort. Previous evidence among MI survivors has shown that poor statin adherence is associated with failure to achieve LDL-C targets, highlighting the importance of assessing statin-specific adherence in future studies [39].

The findings should be interpreted with consideration of potential residual confounding. Factors such as variation in statin therapy intensity, time since myocardial infarction, and physical activity levels were not fully captured in the analysis and may have influenced LDL-C control. Differences in treatment intensity and duration of follow-up, along with the high prevalence of physical inactivity in this cohort, could have contributed to the observed patterns. Further research, particularly using longitudinal designs with more detailed treatment and lifestyle data, is needed to better elucidate their independent effects on LDL-C control.

### Study limitations

The study is cross-sectional, capturing data at a single point in time, which limits the ability to establish causal relationship between identified factors, such as smoking, beta-blocker use, blood pressure control and LDL-C levels. More specifically, the association between beta-blocker use and uncontrolled LDL-C may be affected by confounding by indication, as patients prescribed beta-blockers may have had greater MI severity, residual ischemia or angina, heart failure, arrhythmias, reduced left ventricular function, or other cardiometabolic risk factors that were not fully captured. The absence of longitudinal treatment data also prevented assessment of whether beta-blocker exposure preceded uncontrolled LDL-C. Future longitudinal studies are needed to assess the impact of targeted interventions on LDL-C and other cardiovascular risk factors control. Although the study was conducted at KAUH, one of the largest hospitals in Jordan, the use of a single center sample may limit the generalizability of the findings, as the participants may not fully reflect the demographic and ethnic diversity of the entire population. Additionally, despite recruiting the targeted calculated sample size, increasing the sample size would help yield findings that are more reliable and robust conclusions drawn from the study. Future research conducted at multiple centers in different geographical areas across Jordan, with a much larger number of participants, could improve the representativeness of the study findings. Furthermore, while consecutive sampling minimizes bias by recruiting all available and eligible patients in order, selection bias could still occur, as some patients were unavailable and others were unwilling to participate. Therefore, the findings should be interpreted with caution when generalizing to MI patients managed in other hospitals, primary-care settings, private clinics, or those who do not regularly attend outpatient follow-up. It is worth mentioning that the study lacks a detailed analysis of statin adherence, dosage, and potential side effects, factors that are crucial for understanding LDL-C control. Additionally, not all patients received the same intensity of statin therapy, which may have influenced the study findings. In particular, the reason why some patients were prescribed moderate-intensity statins despite clinical guidelines recommending high-intensity statins for post-MI patients was not clear. This variation in treatment intensity could have contributed to the high prevalence of uncontrolled LDL-C observed in the study. Future research that includes more in-depth analysis of statin therapy, covering adherence rates, intensity levels, dosage variations, and patient-reported side effects is essential for understanding of LDL-C in this high-risk population. Lastly, medication adherence and beliefs were assessed using self-reported questionnaires, which are subject to recall and social desirability biases and overestimation of adherence rates.

## Conclusions

This research demonstrated prevalence of uncontrolled LDL-C in MI patients. Smoking, beta-blocker use, and uncontrolled BP were associated with uncontrolled LDL-C in these patients; however, the association with beta-blocker use should be interpreted cautiously because it may reflect confounding by indication rather than a direct causal effect. To improve health outcomes and decrease the risk of cardiac complications in MI patients with concurrent dyslipidemia, it becomes imperative to develop targeted and effective strategies for managing LDL-C levels. Implementing approaches such smoking cessation counselling, as well as optimizing BP management will be instrumental in achieving this goal.

## Supporting information

S1 File Inclusivity-in-global-research-questionnaire. (DOCX)

S2 File Strobe checklist. (DOC)
